# Identification and characterization of differentially expressed exosomal microRNAs in bovine milk infected with *Staphylococcus aureus*

**DOI:** 10.1186/s12864-019-6338-1

**Published:** 2019-12-05

**Authors:** Shaoyang Ma, Chao Tong, Eveline M. Ibeagha-Awemu, Xin Zhao

**Affiliations:** 1 College of Animal Science and Technology, Northwest A&F University, Yangling, Shaanxi China; 2 Agriculture and Agri-Food Canada, Sherbrooke Research and Development Centre, Sherbrooke, Quebec Canada; 3 Department of Animal Science, McGill University, 21,111 Lakeshore, Ste-Anne-De-Bellevue, Quebec Canada

**Keywords:** Milk, Exosome, Bta-miR-378, Bta-miR-185, *Staphylococcus aureus*

## Abstract

**Background:**

MicroRNAs (miRNAs) in milk-derived exosomes may reflect pathophysiological changes caused by mastitis. This study profiled miRNAs in exosomes from both normal milk and mastitic milk infected by *Staphylococcus aureus* (*S. aureus*). The potential targets for differentially expressed (DE) miRNAs were predicted and the target genes for bta-miR-378 and bta-miR-185 were also validated.

**Results:**

Total RNA from milk exosomes was collected from healthy cows (*n* = 3, the control group) and *S. aureus* infected cows (*n* = 6, the SA group). Two hundred ninety miRNAs (221 known and 69 novel ones) were identified. Among them, 22 known and 15 novel miRNAs were differentially expressed. Target genes of DE miRNAs were significantly enriched in intracellular protein transport, endoplasmic reticulum and identical protein binding. The expression of two miRNAs (bta-miR-378 and bta-miR-185) with high read counts and log_2_ fold changes (> 3.5) was significantly higher in mastitic milk infected with *S. aureus.* One target gene (*VAT1L*) of bta-miR-378 and five target genes (*DYRK1B*, *MLLT3*, *HP1BP3*, *NPR2* and *PGM1*) of bta-miR-185 were validated.

**Conclusion:**

DE miRNAs in exosomes from normal and *S. aureus* infected milk were identified. The predicted targets for two DE miRNAs (bta-miR-378 and bta-miR-185) were further validated. The linkage between the validated target genes and diseases suggested that we should pay particular attention to exosome miRNAs from mastitic milk in terms of milk safety.

## Background

MicroRNAs (miRNAs) are short noncoding (~ 22 nucleotide in length), regulatory RNAs that modulate gene expression at the post-transcriptional level, mostly via binding to perfectly/partially complementary sites at the 3′-UTR of target mRNAs [1]. Among different body fluids, milk contains the highest amount of miRNAs [2]. Milk is an essential source of nutrients to all mammalian offspring. Bovine milk and dairy products have long traditions in human nutrition. In addition to providing nutrition, milk has long been known to protect the infant from infections and to play developmental functions integral to the infant, in which miRNAs are likely to be highly involved [3].

The majority of milk’s miRNAs are transported and protected by the lipid bilayer of extracellular vesicles, predominantly exosomes of about 100 nm in diameter secreted by mammary epithelial cells [4]. Exosomes are cell-derived vesicles that are present in all biological fluids including blood, saliva, urine, amniotic fluid, bronchoalveolar lavage fluid and milk [5, 6]. Milk exosomes have been reported in cows [7], buffalos [8], goats [9], pigs [10], marsupial tammar wallabies [11] and humans [12]. Exosomes protect miRNA molecules from effects of RNase digestion and low pH [13]. Thus, miRNAs in milk exosomes may be transferred into the gastrointestinal tract of infants and likely contribute to infant development and protection against infections [14].

Cells can take up exosomes through a variety of endocytic pathways, including clathrin-dependent endocytosis, clathrin-independent pathways such as caveolin-mediated uptake, macropinocytosis and phagocytosis [15]. Uptake of milk exosomes including their miRNAs has been demonstrated in human colon carcinoma Caco-2 cells and rat intestinal epithelial cell (IEC) IEC-6 cells [16]. Further, orally administered exosomes escaped re-packaging in the intestinal mucosa, and accumulated in liver and spleen. The same group later reported that labelled RNA derived from the milk exosomes was detected in mouse brain, kidney and lung [17]. Porcine milk exosomes promoted IEC proliferation in mice and increased mouse villus height, crypt depth and ratio of villus length to crypt depth of intestinal tissues were associated with miRNA-mediated gene regulatory changes in IECs [18]. In another study, oral delivery of bovine milk exosomes ameliorated experimentally induced arthritis [19]. Correctively, these data suggest that miRNA in milk exosomes can get into the body.

Accumulating evidence suggests that exosomal miRNAs play crucial roles in numerous diseases such as hepatocellular carcinoma [20], breast cancer [21] and Alzheimer’s disease [22]. Secretion of milk exosomes is affected by bacterial infections in mammary glands. *Staphylococcus aureus* (*S. aureus*) is one of the most important etiologic agents for chronic bovine mastitis. Our previous in vitro study showed that 5 miRNAs (miR-2339, miR-21-3p, miR-92a, miR-23a and miR-365-3p) were up-regulated in bovine mammary epithelial cells when challenged with *S. aureus* [23]. In bovine mammary gland infected with *S. aureus*, a total of 77 miRNAs showed significant differences compared to the control group [24]. Previous studies have also investigated milk exosomal miRNAs following *S. aureus* induced bovine mastitis [25, 26]. However, no study has focused on miRNAs in exosomes derived from milk naturally infected with *S. aureus*. More importantly, previous studies focused on the profiling of miRNAs in milk exosomes, without experimental confirmation of the predicted target genes by bioinformatics. In addition, how miRNAs in exosomes affect milk safety has not been considered.

The objective of this study, therefore, was to characterize the miRNA expression profiles comprehensively in exosomes from normal and uninfected milk (the control group) and *S. aureus* infected milk (the SA group) and to predict potential targets for DE miRNAs and explore their possible functions.

## Results

### Identification of *S. aureus* in bovine milk

Based on colony counting and PCR results for *nuc* and bacterial *16S rRNA* genes, 13.95% (42/301) milk samples were infected with *S. aureus.* At the cow level, the infection rate was 31.58% (24/76) (Additional file 8: Table S1)*.*

### Isolation and detection of exosome from bovine milk

Bovine milk exosomes with approximately 100 nm in diameter were observed (Additional file 1: Figure S1a). Exosomes with a particle diameter in the range of 20 nm to 200 nm amounted to 84.1% of the total (Additional file 1: Figure S1b). The expression of CD63 and CD81 on the surface of exosomes was at a positive rate of 72.0 and 77.9%, respectively (Additional file 2: Figure S2).

### Characterization of bovine milk exosomal miRNAs

Average RNA contents of the exosomes from 40 mL of the control or *S. aureus* infected milk samples were 1301 ± 38.7 ng (*n* = 3) and 1223 ± 56.6 ng (*n* = 6), respectively. Bovine milk exosomal RNA contained little or no 28S and 18S ribosomal RNA (data not shown).

**Fig. 1:**
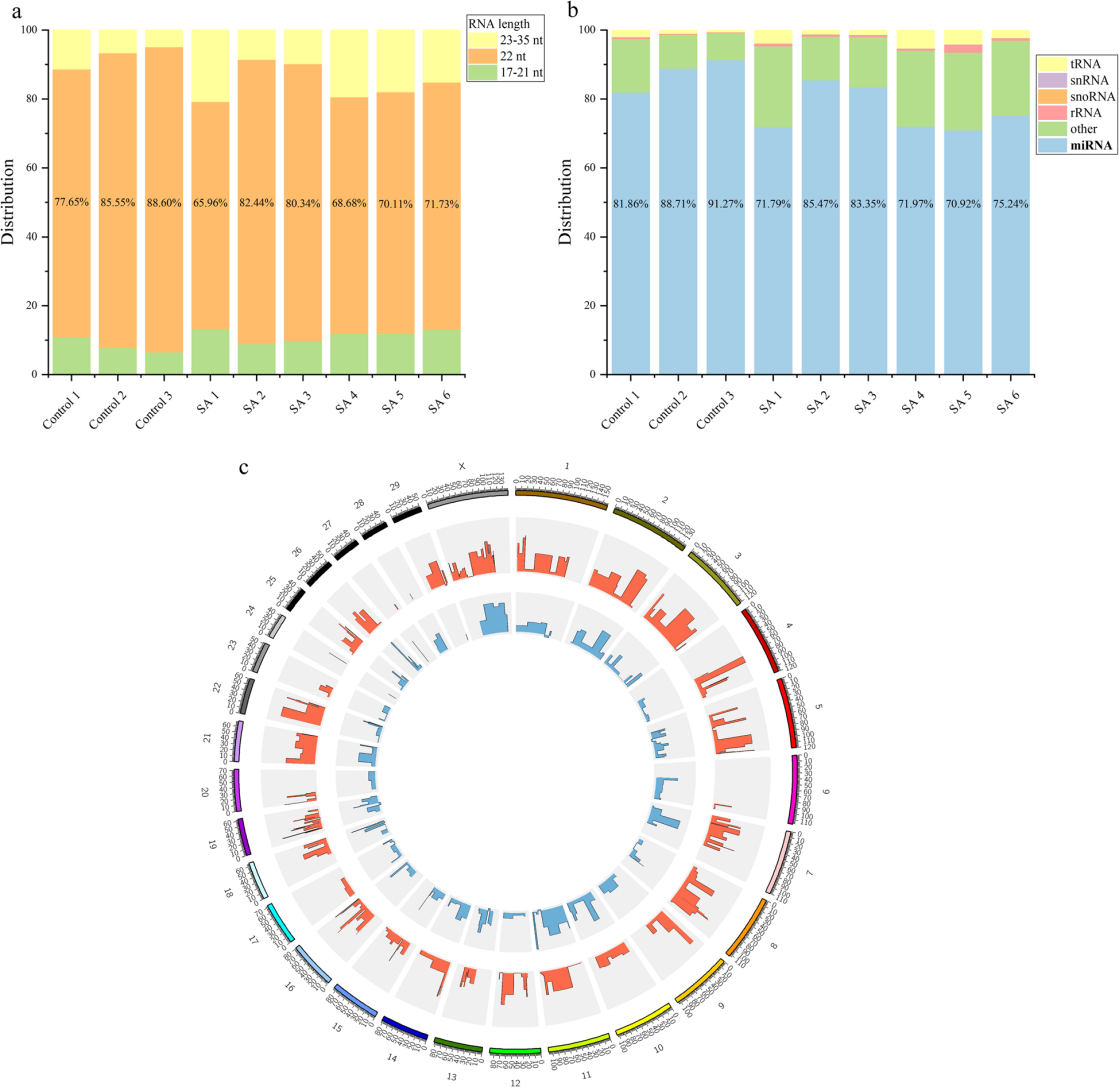
Sequencing data for small RNAs in milk-derived exosomes. (**a**) Length (nt) distribution of the read counts. (**b**) Different categories of small RNAs in 9 studied samples. (**c**) Intuitive map of miRNA distribution across bovine chromosomes (the outermost circle, one unit of the scale stands for one million base-pairs). The middle circle (red lines) represents known miRNAs and the innermost circle (blue lines) represents novel miRNAs. The height of the column is proportional to the expression level and the position of the column corresponds to the miRNA location on chromosome

The total raw read count from the sequencing of 9 libraries was 101,392,712 with an average of 11,265,857 reads per sample. After removing linker reads, reads containing N and poly A/T structure, length-anomalous reads, low-quality reads and reads greater than 35 nt or less than 17 nt, the resulting high quality clean data accounted for 83 to 96% of the original raw read counts. The majority of retained reads were 22 nt in length (Fig. 1a).

Approximately 95% (range from 92.22 to 96.97%) of clean reads were successfully aligned to the bovine Reference Genome (UMD 3.1) (Additional file 9: Table S2). miRNAs are the dominant small RNAs (Fig. 1b, Additional file 10: Table S3).

A total of 221 known and 69 novel miRNAs satisfied the conditions of having at least 1 transcript per million clean tags and were present in a minimum of four libraries. These 290 miRNAs were used for differently expressed (DE) analysis (Additional file 11: Table S4).

Twenty-five miRNAs having > 0.1% of the total read counts in both control and SA groups were regarded as abundantly expressed miRNA (Table 1). Seven most abundantly expressed miRNAs (bta-miR-148a, bta-miR-30a-5p, bta-let-7f, bta-miR-21-5p, bta-miR-26a, bta-let-7a-5p and bta-let-7 g) accounted for 93.80 and 90.91% of the total read counts in the control and SA groups, respectively. Bta-miR-148a had the highest miRNA read counts in both groups. Bta-miR-11_2406 was the most highly expressed novel miRNA, which accounted for 0.139‰ of total read counts (Additional file 11: Table S4b).

**Table 1:** Twenty-five most abundantly expressed miRNAs in bovine milk exosomes

miRNA ID	Control group (*n* = 3)		SA group (*n* = 6)		Overall	
	Mean reads	%	Mean reads	%	Mean reads	%
bta-miR-148a	5,704,941.00	83.444	4,487,889.67	77.659	4,893,573.44	79.809
bta-miR-30a-5p	234,243.67	3.426	260,044.17	4.500	251,444.00	4.101
bta-let-7f	131,014.00	1.926	126,834.67	2.195	128,227.78	2.091
bta-miR-21-5p	93,483.33	1.367	132,236.83	2.288	119,319.00	1.946
bta-miR-26a	102,636.33	1.501	94,022.50	1.627	96,893.78	1.580
bta-let-7a-5p	78,246.67	1.144	83,324.33	1.442	81,631.78	1.331
bta-let-7 g	68,033.00	0.995	69,446.83	1.202	68,975.56	1.125
bta-miR-200a	47,714.67	0.698	59,328.50	1.027	55,457.22	0.904
bta-let-7b	47,204.67	0.690	59,017.83	1.021	55,080.11	0.898
bta-miR-200c	32,256.33	0.472	38,897.00	0.673	36,683.44	0.598
bta-miR-30d	24,246.67	0.355	28,719.33	0.497	27,228.44	0.444
bta-miR-99a-5p	19,972.33	0.292	26,966.17	0.467	24,634.89	0.402
bta-miR-26b	24,645.67	0.360	21,275.50	0.368	22,398.89	0.365
bta-let-7i	18,153.00	0.266	22,036.17	0.381	20,741.78	0.338
bta-miR-27b	19,007.00	0.278	20,082.17	0.348	19,723.78	0.322
bta-miR-151-3p	15,890.00	0.232	18,085.17	0.313	17,353.44	0.283
bta-miR-186	9732.33	0.142	20,533.17	0.355	16,932.89	0.276
bta-miR-200b	11,909.67	0.174	12,660.67	0.219	12,410.33	0.202
bta-let-7c	9782.67	0.143	12,613.83	0.218	11,670.11	0.190
bta-miR-103	6039.00	0.088	10,320.67	0.179	8893.44	0.145
bta-miR-375	7205.33	0.105	9041.00	0.156	8429.11	0.137
bta-miR-182	6910.00	0.101	8220.67	0.142	7783.78	0.127
bta-miR-92a	8571.33	0.125	6102.33	0.106	6925.33	0.113
bta-miR-532	5888.67	0.086	7368.33	0.128	6875.11	0.112
bta-miR-423-5p	4426.67	0.064	7251.67	0.125	6310.00	0.103

A higher number of known miRNAs were located on Chr X (36 miRNAs), Chr 19 (29 miRNAs), and Chr 21 (27 miRNAs), while the highest number of novel miRNAs were located on Chr 5 (15 miRNAs) (Fig. 1c).

### DE miRNAs in exosomes between the control and *S. aureus* infected milk

**Fig. 2:**
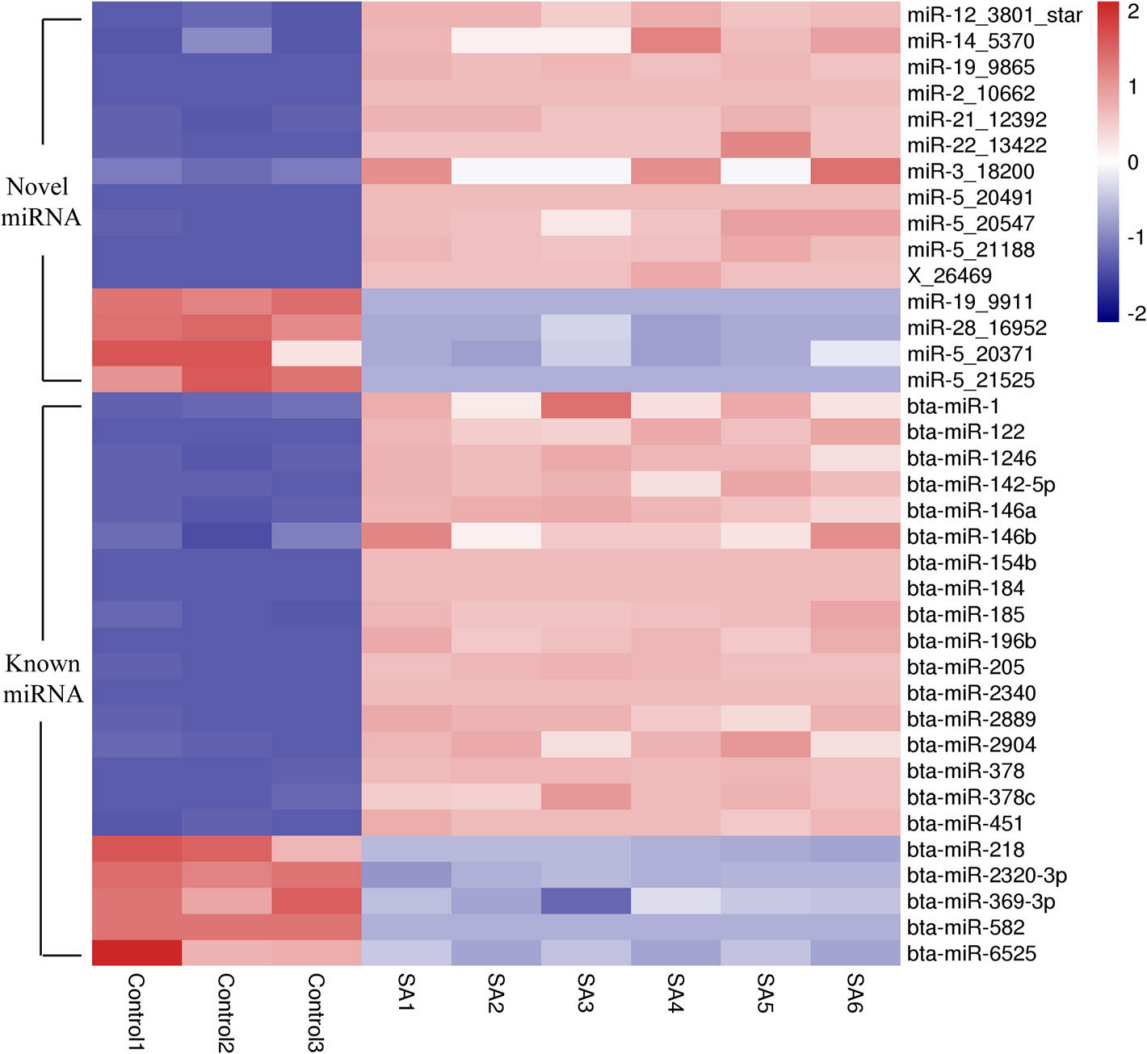
Heat map of DE miRNAs expression profile. The intensity of each colour indicates the log_2_ (the miRNA expression in each sample/ the average of miRNA expression across all 9 samples). Red colour pixels indicate an increased abundance of miRNA in the indicated samples whereas blue pixels indicate decreased miRNA levels in log_2_ scale

Thirty seven miRNAs (twenty two known and fifteen novel) were significantly differentially expressed (*p* < 0.05) between the control and the SA groups. Out of these, twenty-eight miRNAs were significantly (*p* < 0.05) up-regulated, whereas nine miRNAs were significantly (*p* < 0.05) down-regulated (Fig. 2). Notably, four miRNAs (bta-miR-2_10662, bta-miR-5_20491, bta-miR-184 and bta-miR-2340) were only expressed in the SA group, while one miRNA (bta-miR-5_21525) was only expressed in the control group (Table 2). Furthermore, three known (bta-miR-185, bta-miR-2904 and bta-miR-378) and eight novel (bta-miR-12_3801, bta-miR-14_5370, bta-miR-21_12392, bta-miR-22_13422, bta-miR-3_18200, bta-miR-5_20547, bta-miR-5_21188 and bta-miR-X_26469) miRNAs were highly expressed (log2foldchange > 3) in the SA group as compared to the control group.

**Table 2:** DE miRNAs between the control group and the SA group

miRNA ID	Log2fold change^a^	*p*-value
Novel miRNAs		
bta-miR-12_3801	3.002	2.48 × 10^−4^
bta-miR-14_5370	3.807	1.14 × 10^−2^
bta-miR-19_9865	2.663	2.94 × 10^−2^
bta-miR-19_9911	−1.585	3.83 × 10^−2^
bta-miR-2_10662	$	5.15 × 10^−5^
bta-miR-21_12392	4.644	7.64 × 10^−4^
bta-miR-22_13422	4.170	1.22 × 10^−3^
bta-miR-28_16952	−1.280	3.15 × 10^−2^
bta-miR-3_18200	3.807	1.22 × 10^−2^
bta-miR-5_20371	−1.078	2.87 × 10^−2^
bta-miR-5_20491	$	1.20 × 10^−2^
bta-miR-5_20547	4.000	2.27 × 10^− 3^
bta-miR-5_21188	4.000	2.10 × 10^− 3^
bta-miR-5_21525	$$	1.75 × 10^−3^
bta-miR-X_26469	5.170	2.93 × 10^−6^
Known miRNAs		
bta-miR-1	1.926	2.40 × 10^−2^
bta-miR-122	1.838	3.17 × 10^−5^
bta-miR-1246	2.140	5.35 × 10^−3^
bta-miR-142-5p	1.864	1.49 × 10^−2^
bta-miR-146a	1.978	3.01 × 10^−2^
bta-miR-146b	1.482	4.46 × 10^−2^
bta-miR-154b	2.848	1.90 × 10^−2^
bta-miR-184	$	2.31 × 10^−8^
bta-miR-185	3.585	3.74 × 10^−2^
bta-miR-196b	2.841	4.92 × 10^−2^
bta-miR-205	2.883	2.05 × 10^−2^
bta-miR-218	−1.078	3.40 × 10^−2^
bta-miR-2320-3p	−2.322	4.02 × 10^−2^
bta-miR-2340	$	1.21 × 10^−2^
bta-miR-2889	2.816	1.56 × 10^−2^
bta-miR-2904	3.484	1.66 × 10^−3^
bta-miR-369-3p	−1.625	1.01 × 10^−2^
bta-miR-378	4.044	1.46 × 10^−3^
bta-miR-378c	2.193	9.65 × 10^−3^
bta-miR-451	1.554	1.85 × 10^−3^
bta-miR-582	−2.000	7.50 × 10^−3^
bta-miR-6525	−2.585	2.37 × 10^−2^

^a^ Values represent the amount of expression of the miRNA in the SA group divided by the amount in the control group, followed by a base-2 logarithmic value

$ miRNA only expressed in the SA group

$$ miRNA only expressed in the control group

### Predicted target genes of known DE miRNAs and GO and KEGG pathway annotations

Twenty-two known DE miRNAs were predicted to target 2678 genes (Additional file 12: Table S5). Bta-miR-185 had the highest number of target genes (515 genes) (Additional file 3: Figure S3), while the *MTMR3* gene was the most popular target for DE miRNAs (targeted by 8 DE miRNAs). Other common target genes for DE miRNAs were *USP12*, *SYT13*, *PDHA1*, *FRMD8*, *KLHL29*, *MCAT*, *ABAT*, *CHFT8* and *CELF3* (each targeted by 6 DE miRNAs).

**Fig. 3:**
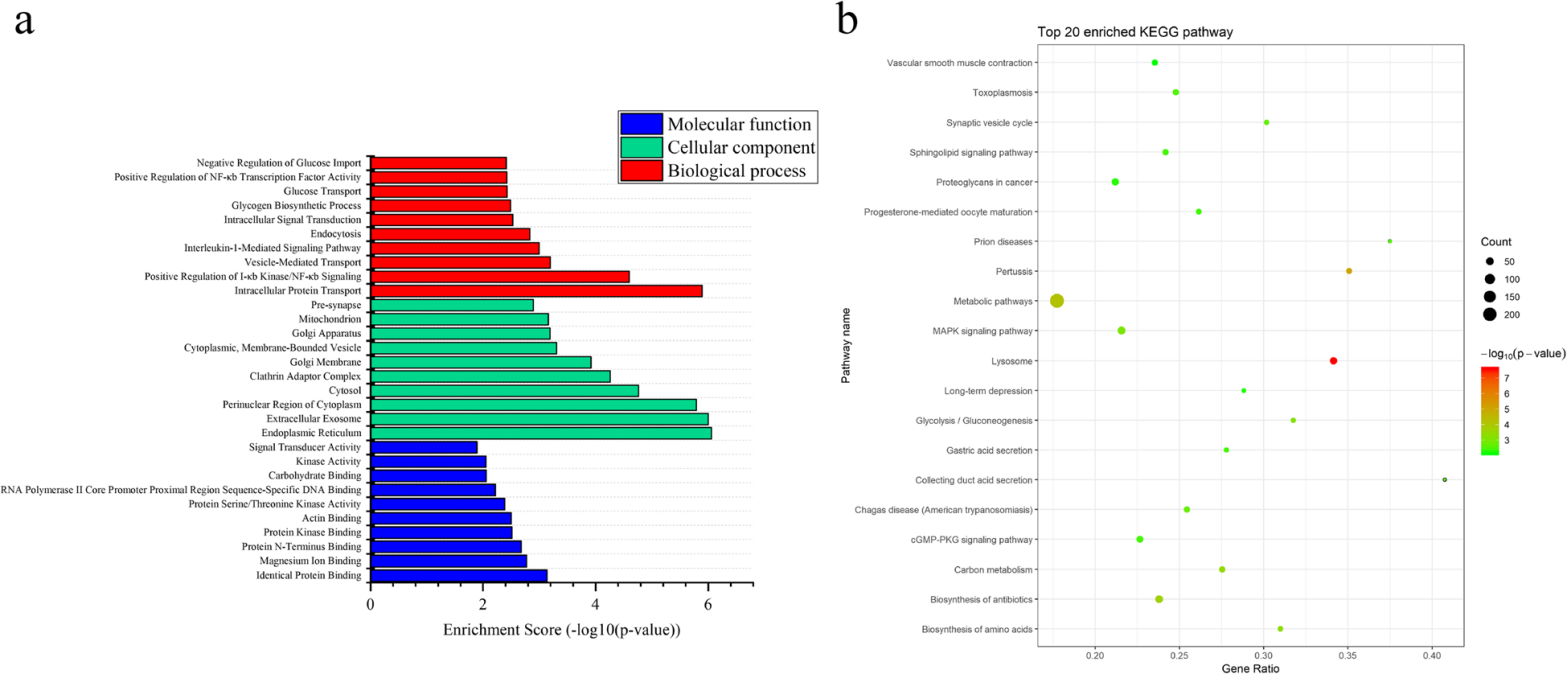
GO and KEGG analyses of 22 known DE miRNA. **a** Top 10 GO terms of 22 known DE miRNAs target genes in each three GO categories (biological process, cellular component, and molecular function). Enrichment score is presented as -log_10_(*p*-value). **b** Top 20 enriched KEGG pathways for target genes of 22 known DE miRNAs. The sizes of the dots represent the counts of genes. The gene ratio indicates the ratio between the number of target genes associated with a KEGG term and the total number of genes in the KEGG term

Target genes of DE miRNAs were significantly (*p* < 0.05) enriched in 121 GO terms (63 biological process GO terms, 34 cellular component GO terms and 24 molecular function GO terms) (Additional file 13: Table S6). The most enriched biological process, cellular component and molecular function GO terms were intracellular protein transport (*p* = 1.29 × 10^− 6^), endoplasmic reticulum (*p* = 8.79 × 10^− 7^) and identical protein binding (*p* = 7.28 × 10^− 4^), respectively (Fig. 3a). Moreover, 49 KEGG pathways were significantly enriched for the target genes of DE miRNAs (Additional file 14: Table S7). The lysosome pathway (*p* = 2.73 × 10^− 8^) was the most significantly enriched KEGG pathway (Fig. 3b).

### Target genes for bta-miR-378 and bta-miR-185 were validated

**Fig. 4:**
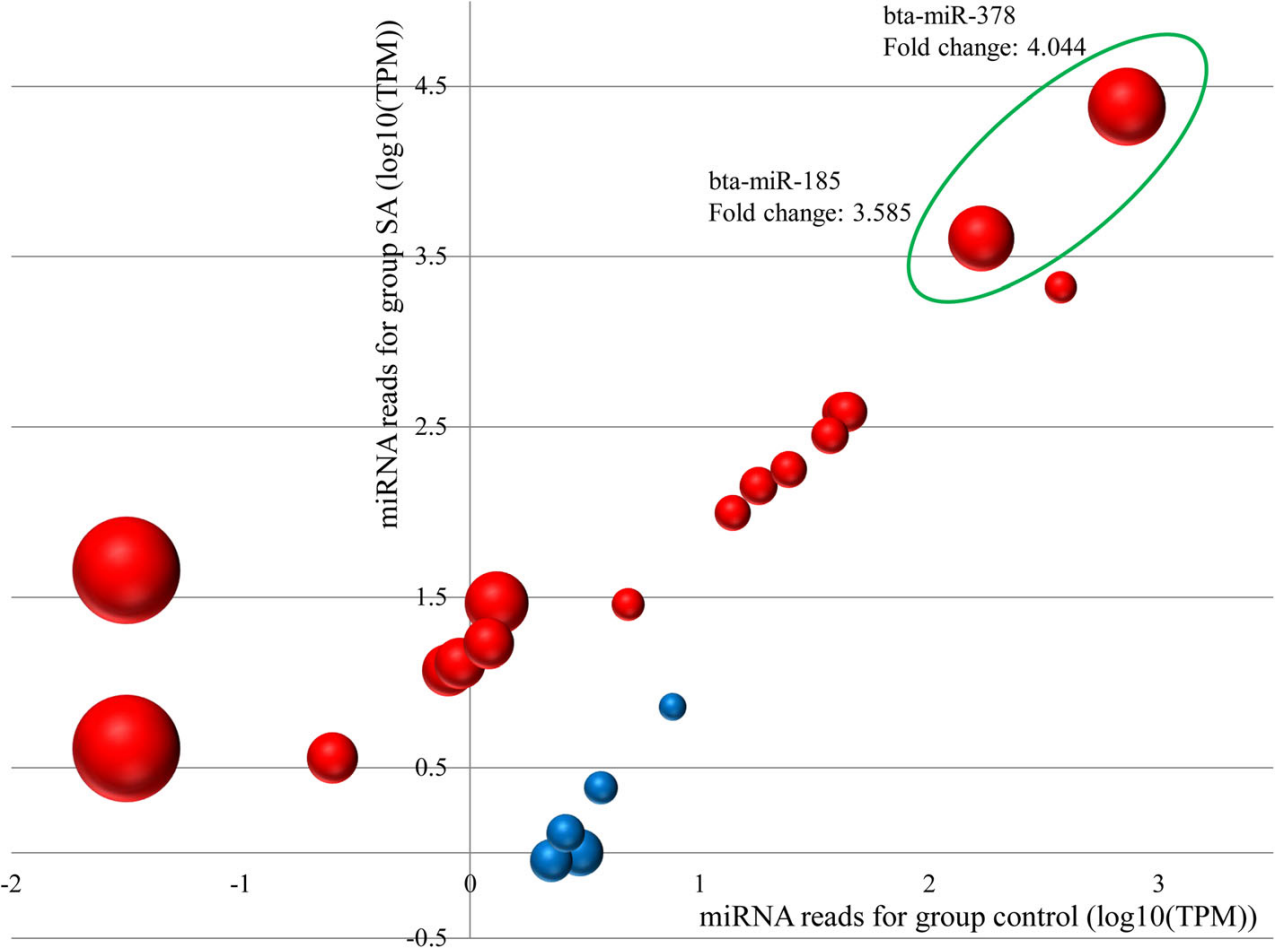
The fold change and read counts (log_10_(TPM)) of DE miRNAs in the control and SA groups. The size of bubble stands for the DE miRNA fold change. The up- and down-regulated miRNAs are colored in red and blue, respectively. Bta-miR-378 and bta-miR-185 are shown inside of the green ellipse

Read counts of DE miRNAs showed that bta-miR-378, bta-miR-185 and bta-miR-146b were 3 top DE miRNAs in the SA group as compared to the control group. Considering the potential importance based on read counts and log2foldchange values, the potential targets for bta-miR-378 and bta-miR-185 were further validated (Fig. 4).

A total of 441 and 814 target genes were predicted for bta-miR-378 and bta-miR-185 by TargetScan and miRanda programs, respectively (Additional file 15: Table S8). Among them, 8 and 23 genes were predicted by both programs, and considered as more plausible targets of bta-miR-378 and bta-miR-185, respectively (Table 3, Additional file 4: Figure S4).

**Table 3:** Plausible target genes of bta-miR-378 and bta-miR-185 predicted by both TargetScan and miRanda programs

miRNA	Predicted target gene	Gene name
bta-miR-378	UBE2W	ubiquitin-conjugating enzyme E2W (putative)
	TCF12	transcription factor 12
	TLK2	tousled-like kinase 2
	TBX6	T-box 6
	VAT1L	vesicle amine transport 1-like
	AQP3	aquaporin 3 (Gill blood group)
	RNF144B	ring finger protein 144B
	PDIA4	protein disulfide isomerase family A, member 4
bta-miR-185	PAK7	p21 protein (Cdc42/Rac)-activated kinase 7
	CAPZB	capping protein (actin filament) muscle Z-line, beta
	KDM2A	lysine (K)-specific demethylase 2A
	PHYHIP	phytanoyl-CoA 2-hydroxylase interacting protein
	NFATC3	nuclear factor of activated T-cells, cytoplasmic, calcineurin-dependent 3
	E2F6	E2F transcription factor 6
	CHMP7	charged multivesicular body protein 7
	DGKI	diacylglycerol kinase, iota
	CYP4V2	cytochrome P450, family 4, subfamily V, polypeptide 2
	SF1	splicing factor 1
	FOSL2	FOS-like antigen 2
	CABP4	calcium binding protein 4
	NR1D1	nuclear receptor subfamily 1, group D, member 1
	HP1BP3	heterochromatin protein 1, binding protein 3
	DYRK1B	dual-specificity tyrosine-(Y)-phosphorylation regulated kinase 1B
	MLLT3	myeloid/lymphoid or mixed-lineage leukemia (trithorax homolog, Drosophila); translocated to, 3
	STX4	syntaxin 4
	SGMS1	sphingomyelin synthase 1
	NPR2	natriuretic peptide receptor B
	SPATA2	spermatogenesis associated 2
	AGFG1	ArfGAP with FG repeats 1
	PGM1	phosphoglucomutase 1
	SDHC	succinate dehydrogenase complex

**Fig. 5:**
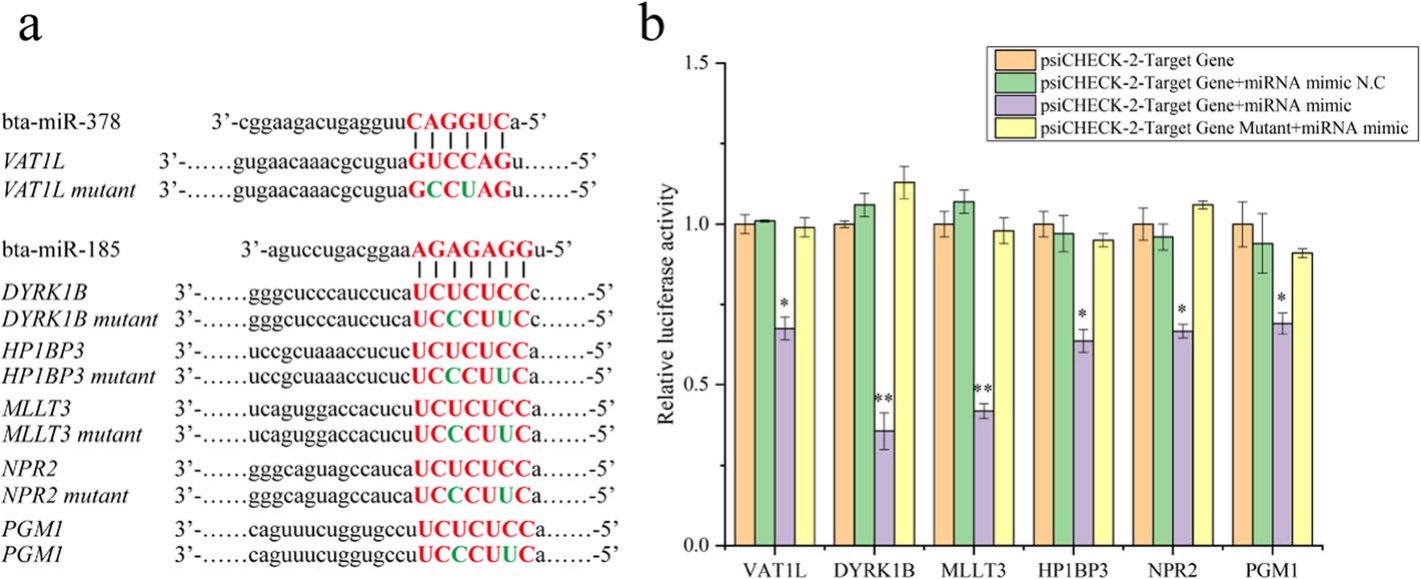
Detection of bta-miR-378 and bta-miR-185 target genes. **a** Binding sites of bta-miR-378 and bta-miR-185 and their target gene vectors. The seed region is indicated by red bases and the green bases are the inserted mutations. **b** Relative luciferase activities of target genes for bta-miR-378 and bta-miR-185. The values represent the mean ± SD of three independent experiments. *p < 0.05, **p < 0.01, N. C, negative control

The binding sites for bta-miR-378 and bta-miR-185 in the 3′-UTR of commonly predicted target genes were analyzed by bioinformatics methods (microrna.org and TargetScan) (Additional file 16: Table S9). To biochemically confirm the in silico predicted targets, the 3′-UTRs of predicted candidate mRNAs were cloned into a dual luciferase vector. The luciferase activity of the psiCHECK-2 vector with 3′-UTR of *VAT1L* was strongly inhibited by bta-miR-378 (*p* < 0.05) (Additional file 5: Figure S5). Similarly, luciferase activities of psiCHECK-2 vectors with 3′-UTR of *DYRK1B*, *MLLT3*, *HP1BP3*, *NPR2* or *PGM1* were significantly down-regulated by bta-miR-185 (*p* < 0.05) (Additional file 6: Figure S6). To validate these results, the miRNA target sites in the 3′-UTRs of *VAT1L*, *DYRK1B*, *MLLT3*, *HP1BP3*, *NPR2* and *PGM1* were mutated (Fig. 5a). After the mutation, transfection of the miRNA mimics (bta-miR-378 or bta-miR-185) did not change the luciferase activities (Fig. 5b). These results suggested that *VAT1L* was the target of bta-miR-378, while *DYRK1B*, *MLLT3*, *HP1BP3*, *NPR2* and *PGM1* were targets of bta-miR-185.

## Discussion

Milk provides important nutrients that are of benefit to most people throughout life. Due to the direct effects of the protein, fat, lipid, vitamin, and mineral fractions, milk has a specific growth promoting effect in children in both developing and developed countries [27].

Pasteurization is widely used in commercial milk production and it destroys all known pathogens and most of the spoilage bacteria in raw milk. Nowadays, there is compelling evidence that milk exosomes are retained in pasteurized commercial milk [17] and reach the systemic circulation and tissues of the human milk consumer [28]. Furthermore, pasteurization did not affect the profile expression of miRNA in bovine milk [29]. Bovine milk exosomes miRNAs which resist the harsh conditions in the gastrointestinal tract [30] are taken up via receptor mediated endocytosis by intestinal epithelial cells [16] and vascular endothelial cells [31]. More importantly, in vivo studies confirmed that milk exosomes miRNAs could reach distant tissues [19] and human plasma [32].

In our study, two miRNAs (bta-miR-378 and bta-miR-185) with high read counts were up-regulated significantly in exosomes from bovine milk infected by *S. aureus*. These two miRNAs have been reported to be associated with health. MiRNA-378 facilitates the development of hepatic inflammation and fibrosis [33]. In addition, expression of the miR-378 was reported to promote tumor growth [34]. MiR-185-5p may inhibit ameloblast and osteoblast differentiations and result in cleidocranial dysplasia [35], and promotes lung epithelial cell apoptosis [36]. How these miRNAs affect health parameters is not clear and it is plausible that their target genes are involved.

Consistent with two previous studies [25, 26], the expression level of bta-miR-148a was the highest among all milk-derived exosomal miRNAs in our study. In these two previous studies, the miRNAs with the greatest differences in expression in milk-derived exosomes after *S. aureus* infection were bta-miR-142-5p [25] and bta-miR-223 [26], respectively. While the expression level of bta-miR-142-5p was also up-regulated significantly in our study, it was not the most differentially expressed one. In addition, expression of bta-miR-223 was not significantly changed in our study. These discrepancies between our study and the other two studies could be due to the fact that exosomes were isolated from mastitic milk naturally infected with *S. aureus* in this study, while the other two studies used milk samples from the mammary gland challenged with *S. aureus*.

We have functionally validated *VAT1L* as a target gene of bat-miR-378. Through a network-based analysis of three independent schizophrenia genome-wide association studies, Chang et al. reported that *VAT1L* may be one of the genes associated with schizophrenia [37]. In addition, *DYRK1B*, *HP1BP3*, *MLLT3*, *NPR2* and *PGM1* were identified as the target genes of bta-miR-185 in our study. Surprisingly, deficiency of these target genes also leads to a variety of diseases. DYRK1B belongs to the Dyrk family of proteins, a group of evolutionarily conserved protein kinases that are involved in cell differentiation, survival, and proliferation [38]. Mutations in *DYRK1B* were associated with a clinical phenotype that is characterized by central obesity, hypertension, type II diabetes and early-onset coronary artery disease [38]. HP1BP3 was identified as a novel modulator of cognitive aging and HP1BP3 protein levels were significantly reduced in the hippocampi of cognitively impaired elderly humans relative to cognitively intact controls [39]. Targeted knockdown of *HP1BP3* in the hippocampus induced cognitive deficits [40]. *MLLT3* gene is required for normal embryogenesis in mice, and an *MLLT3* null mutation caused perinatal lethality [41]. A loss-of-function mutation of the *AF9/MLLT3* gene was hypothesized to relate to neuromotor development delay, cerebellar ataxia and epilepsy [42]. Homozygous inactivating mutations of *NPR2* caused a severe skeletal dysplasia, acromesomelic dysplasia and Maroteaux type [43]. PGM1 deficiency has been described in a patient with myopathy and exercise induced hypoglycemia [44, 45]. PGM1 deficiency causes a non-neurological disorder of glycosylation as well as a rare muscular glycolytic defect [46].

In addition to bta-miR-378 and bta-miR-185, several other miRNAs were also differentially expressed, including miR-1, miR-122, miR-1246, miR-142-5p, miR-146a, miR-154, miR-184, miR-196 and miR-205. They were also associated with a variety of human diseases. For example, circulating miR-122 is strongly associated with the risk of developing metabolic syndrome and type II diabetes [47]. MiR-196 was overexpressed in the inflammatory intestinal epithelia of individuals with Crohn’s disease [48]. While it is beyond the scope of this study to confirm the linkage between discussed miRNAs and health parameters, the above discussion about increased expression of certain miRNAs in exosomes from *S. aureus* infected milk argues strongly to be vigilant about the safety of mastitis milk, even after pasteurization.

## Conclusions

In conclusion, we characterized the miRNA profiles in exosomes derived from control and *S. aureus* infected bovine milk, and 37 miRNAs (22 known and 15 novel) were significantly differentially expressed between the control group and SA group. This is the first report of functional validation of *VAT1L*, and *DYRK1B*, *MLLT3*, *HP1BP3*, *NPR2* and *PGM1* as target genes for bta-miR-378 and bta-miR-185, respectively. Finally, the potential safety hazards of mastitic milk were discussed, in the context of miRNAs within milk exosomes.

## Methods

### Milk sample collection and bacteria identification

Milk samples from seventy-six 3- to 4-year-old Holstein cows in the middle stage of lactation from 4 dairy farms (the Shaanxi Academy of Agricultural Sciences Farm, the farm of Delikang Dairy Co., Ltd., the Duzhai dairy farm and the Cuidonggou dairy farm) in Shaanxi Province were collected for this study with the approval of the Animal Use and Care Committee of Northwest A&F University (NWAFAC3751). Milk samples from all four quarters of each cow were aseptically collected and stored at − 80 °C.

To select the samples for the control and the SA groups, 100 μL of each milk sample were plated on the Plate Count Agar (BD Diagnostics, Sparks, MD, USA) and incubated at 32 °C for 48 h. The control group samples (*n* = 3) were randomly selected from the samples among which the colony count was zero. The milk samples with colony counts more than 1000 were marked as samples with bacterial infection for further detection. To rule out the interference caused by *Escherichia coli* (*E. coli*) infection for subsequent experiments, samples were cultured on the BactiCard™ *E. coli* (Thermo Oxoid Remel, Lenexa, USA). The milk samples without *E. coli* infection were selected for the identification of *S. aureus* by the Baird-Parker agar (Oxoid, Basingstoke, Hampshire, UK) as described previously [49]. Briefly, aliquots of individual milk samples were added to an equal volume of a double-strength enrichment broth (a trypticase soy broth supplemented with 10% NaCl and 1% sodium pyruvate) (Oxoid, Basingstoke, Hampshire, UK). After 24 h incubation at 35 °C, the enrichment broth was streaked onto the Baird-Parker (Oxoid) agar containing 30% egg yolk with 1% tellurite (Oxoid) and onto the phenol red mannitol salt agar plates. Following 48 h incubation at 35 °C, the colonies on the plates were counted, and one to three presumptive staphylococcal colonies from each plate were transferred to trypticase soy agar plates. Yellow colored colonies from the phenol red mannitol salt agar plates were assumed to be *S. aureus*. Further identification of these presumptive staphylococcal colonies was first based on conventional methods including Gram stain staining, colony morphology, a catalase test and a coagulase test with rabbit plasma. The culture result was further confirmed by using a polymerase chain reaction (PCR) assay targeting *S. aureus*-specific region of the thermonuclease gene (*nuc*) [49] and bacterial *16S rRNA* genes. The samples with *nuc* positive *S. aureus*, which was also confirmed by sequencing the PCR products of *16S rRNA* genes, were selected for the SA group (*n* = 6).

### Preparation and purification of milk exosomes

Milk exosomes from the SA group (*n* = 6) and the control group (*n* = 3) were isolated by differential centrifugation as described previously [50]. Briefly, milk samples were centrifuged at 5000×g for 60 min at 4 °C to remove milk fat and milk somatic cell. For the removal of casein and other cell debris, skimmed milk samples were subjected to three successive centrifugations at 4 °C for 1 h each at 12,000×g, 35,000×g and 70,000×g (Beckman Coulter, USA). The whey was collected and centrifuged at 135,000×g at 4 °C for 90 min (Beckman Coulter) to remove large particles and micro-vesicles. The supernatant was carefully collected and filtered through a 0.22 μm syringe-driven filter unit (Merck KGaA, Darmstadt, Germany). The percolate was collected and centrifuged at 150,000×g for 90 min at 4 °C (Beckman Coulter). The exosome pellet was re-suspended in 1 mL sterile PBS and filtered through a 0.22 μm syringe-driven filter unit (Merck KGaA). Finally, exosomes were stored in aliquots of 200 μL at − 80 °C until being used.

### Identification of bovine milk exosomes

Dynamic light scattering analysis was used for analyzing nanoparticle sizes. Aliquot of 200 μL stored exosomes was diluted to 1 mL volume with sterile PBS stored on ice. The exosome solution was slowly injected into the sample cell of the Malvern Zetasizer Nano ZS90 (Malvern Panalytical Ltd., United Kingdom) system and measurements were taken according to manufacturer’s instructions.

For transmission electron microscopy (TEM), milk exosomes were fixed in 3% (w/v) glutaraldehyde and 2% paraformaldehyde in a cacodylate buffer, pH 7.3. The fixed exosomes were then applied to a continuous carbon grid and negatively stained with 2% uranyl acetate. The samples were examined with a HT7700 transmission electron microscope (HITACHI, Japan).

Exosome marker proteins (CD63 and CD81) were detected via flow cytometry (Accuri™ C6, BD Biosciences, USA) using anti-CD63 (BD Biosciences, USA) and anti-CD81 (BD Biosciences, USA) antibodies, according to manufacturer’s protocols.

### Extraction of total RNA from bovine milk exosomes

Total RNA was extracted from bovine milk exosomes using the Trizol reagent (TAKARA, Japan) according to manufacturer’s protocol and dissolved in RNase free water. Quality and quantity of RNA was examined using a NanoDrop 2000/2000C (Thermo Fisher Scientific, Waltham, MA, USA) and integrity was detected using agarose gel electrophoresis.

### MiRNA library preparation and sequencing

Deep sequencing was performed on all 9 individual samples. For each library, 1 μg of high-quality RNA per sample was used as the input material for a small RNA library construction using the NEXTflex™ Small RNA Sequencing Kit V3 (Illumina, San Diego, CA) according to the manufacturer’s instruction. Small RNA libraries were gel purified and pooled together in equimolar concentrations and subjected to 50 bp single read sequencing on an Illumina HiSeq 2500 system (Illumina, San Diego, CA). Read quality (adaptor removal and size selection) was assessed using FastQC v0.11.5 (http://www.bioinformatics.babraham.ac.uk/projects/fastqc/) and the cutadapt [51].

### Known miRNAs identification and novel miRNAs discovery

Identification of known miRNAs was performed with miRBase v21 (http://www.mirbase.org/) [52], while novel miRNA discovery was achieved with miRDeep2 v2.0.0.8 (https://github.com/rajewsky-lab/mirdeep2) [53]. The core and quantifier modules of miRDeep2 were applied to discover novel miRNAs in the pooled dataset of all the libraries while the quantifier module was used to profile the detected miRNAs in each library. The amount of miRNA expression was calculated by the transcript per million (TPM) metric, which is calculated as number of reads per miRNA alignment/number of reads of the total sample alignment× 10^6^. MiRDeep2 score > 1 was used as a cuff point for identification of novel miRNAs. Subsequently, a threshold of ≥1 TPM of total reads and present in ≥4 libraries was applied to remove lowly expressed miRNAs. MiRNAs meeting these criteria were further used in downstream analyses including differential expression analyses.

### Differential miRNA expression and predicted target genes

DE miRNAs were detected with DeSeq2 (v1.14.1) (https://bioconductor.org/packages/release/bioc/html/DESeq2.html) [54]. Following normalization, miRNAs read counts in the SA group were compared with corresponding values in the control group. Significant DE miRNAs between the control and the SA groups were defined as having a Benjamini and Hochberg [55] corrected *p*-value< 0.05.

In order to investigate the potential functions of DE miRNAs, their target genes were predicted using the miRanda algorithm [56]. Predicted target genes with tot scores above 150 and tot energy below − 15 were further used for pathway analyses. The Database for Annotation, Visualization and Integrated Discovery (DAVID) was used to perform Gene Ontology (GO) and Kyoto Encyclopedia of Genes and Genomes (KEGG) pathway annotations of their target genes [57].

### Bta-miR-378 and bta-miR-185 target genes validation and function analysis

Among all DE miRNAs, two miRNAs (bta-miR-378 and bta-miR-185) with high expression levels and the highest log2fold change values between the two groups were further investigated. Target genes of bta-miR-378 and bta-miR-185 were predicted by Target Scan 7.0 (http://www.targetscan.org), miRDB (http://www.mirdb.org/miRDB) and miRanda (http://www.microrna.org/microrna/home.do). Then, 3′-UTRs of target gene transcripts for bta-miR-378 and bta-miR-185 were amplified with specific primers (Additional file 17: Table S10). Furthermore, the seed regions in the 3′-UTRs of the genes were mutated with mutagenic primers by using overlapping extensions (Additional file 17: Table S10). The wild-type and mutated 3′-UTRs were sub-cloned into the restriction endonuclease *NotI* and *XhoI* site of the psiCHECK-2 vector (Promega, Madison, WI, USA).

The HEK293-T cell line (ATCC, Manassas, VA, USA) was used for transfection. The bta-miR-378 mimic (5’ACUGGACUUGGAGUCAGAAGGC3’), bta-miR-185 mimic (5’UGGAGAGAAAGGCAGUUCCUGA3’) and a miRNA mimic negative control (N.C, 5’UUGUACUACACAAAAGUACUG3’) were synthesized by GenePharma (Shanghai, China). Transfection of the miRNA mimic and psiCHECK-2 was achieved using the Lipofectamine® 3000 reagent (Invitrogen, USA) according to the manufacturer’s instructions (Additional file 7: Figure S7). Twenty-four hours after transfection, the medium was changed and cells were grown for an additional 24 h before the luciferase assay.

Firefly and Renilla luminescent signals arising from transfected cells were quantified according to the manufacturer’s instructions using a Dual Luciferase assay system (Promega) with a Multilabel Counter luminometer (Varioskan Flash, Thermo Fisher Scientific). Renilla luciferase activities to firefly luciferase activities in cells transfected with an empty psiCHECK-2 vector without a 3′-UTR fragment was set to 100%. The experiment was repeated 3 times.

### Statistical analysis

Data were analyzed with the SPSS 17.0 software (SPSS Inc., Chicago, IL, USA). The statistical significance between experimental groups was analyzed using One-Way ANOVA. *p* < 0.05 and *p* < 0.01 were defined to be statistically significant and extremely significant, respectively.

## Supplementary information

**Additional file 1: Figure S1.** Bovine milk exosomes. (a) Electron microscopy images of exosomes isolated from bovine milk. (b) Particle size analysis of exosomes isolated from bovine milk by ultracentrifugation.

**Additional file 2: Figure S2.** Expression of CD63 and CD81 on exosome surfaces by flow cytometry.

**Additional file 3: Figure S3.** The number of target genes for 22 known DE miRNAs predicted by using miRanda.

**Additional file 4: Figure S4.** Number of predicted target genes of bta-miR-378 (A) and bta-miR-185 (B) by TargetScan (blue) and miRanda (red) programs.

**Additional file 5: Figure S5.** Relative luciferase activities of target genes for bta-miR-378. The values represent the mean ± SD of three independent experiments. *p < 0.05, **p < 0.01, N.C. stands for negative control.

**Additional file 6: Figure S6.** Relative luciferase activities of target genes for bta-miR-185. The values represent the mean ± SD of three independent experiments. *p < 0.05, **p < 0.01, N.C. stands for negative control.

**Additional file 7: Figure S7.** Verification of in silico predicted bta-miR-378 and bta-miR-185 targets with a dual luciferase assay. HEK293 cells were transfected with bta-miR-378/bta-miR-185 mimics together with a vector carrying the firefly luciferase gene (*hluc*+) as well as the Renilla luciferase gene (*hRluc*) fused to the 3′-UTR fragment containing either the predicted bta-miR-378/bta-miR-185 binding site or the mutated seed sequence. The assays were performed 48 h later, and the ratio of Renilla luciferase activities to firefly luciferase activities in cells transfected with an empty expression vector without 3′-UTR fragment of the target gene was set to 100%.

**Additional file 8: Table S1.** Results for bacterial culture and nuc detection.

**Additional file 9: Table S2.** Alignment rates of clean reads with the bovine Reference Genome (UMD 3.1).

**Additional file 10: Table S3.** Classification statistics for small RNA sequences.

**Additional file 11: Table S4.** a. Read counts and read counts expressed as transcript per million (TPM) for known miRNAs. b. Read counts and read counts expressed as transcript per million (TPM) for novel miRNAs.

**Additional file 12: Table S5.** Predicted target genes of DE miRNAs.

**Additional file 13: Table S6.** a. Gene ontologies (biological process) enriched for target genes of 22 known DE miRNAs. b. Gene ontologies (cellular component) enriched for target genes of 22 known DE miRNAs. c. Gene ontologies (molecular function) enriched for target genes of 22 known DE miRNAs.

**Additional file 14: Table S7.** KEGG pathways enriched for the target genes of DE miRNAs.

**Additional file 15: Table S8.** a. Predicted target genes of bta-miR-378 by TargetScan and miRanda. b. Predicted target genes of bta-miR-185 by TargetScan and miRanda.

**Additional file 16: Table S9.** a. The binding sites between bta-miR-378 and 3′-UTR of target genes. b. The binding sites between bta-miR-185 and 3′-UTR of target genes.

**Additional file 17: Table S10**. PCR primers.

## Data Availability

The datasets generated and analyzed during the current study are available in the NCBI Sequence Read Archive (BioProject No.: PRJNA589206, SRA Accession: SRR10439412, SRR10439411, SRR10439410, SRR10439409, SRR10439408, SRR10439407, SRR10439406, SRR10439405 and SRR10439404).

## Abbreviations

bta: *Bos taurus*
Chr: Chromosome
DAVID: Database for Annotation, Visualization and Integrated Discovery
DE: Differentially expressed
DMEM: Dulbecco’s modified eagle medium
FBS: Fetal bovine serum
GO: Gene Ontology
IEC: Intestinal epithelial cell
KEGG: Kyoto Encyclopedia of Genes and Genomes
miRNA: microRNA
PBS: Phosphate buffer saline
*S. aureus*: *Staphylococcus aureus*
snoRNA: small nucleolar RNA
snRNA: small nuclear RNA
TPM: Transcript per million
UTR: Untranslated region
