# Supplementary figures and images for: Identification and characterization of differentially expressed exosomal microRNAs in bovine milk infected with Staphylococcus aureus
Source: BMC Genomics. 2019 Dec 5;20:934. doi: 10.1186/s12864-019-6338-1 (PMC6896338; doi:10.1186/s12864-019-6338-1)

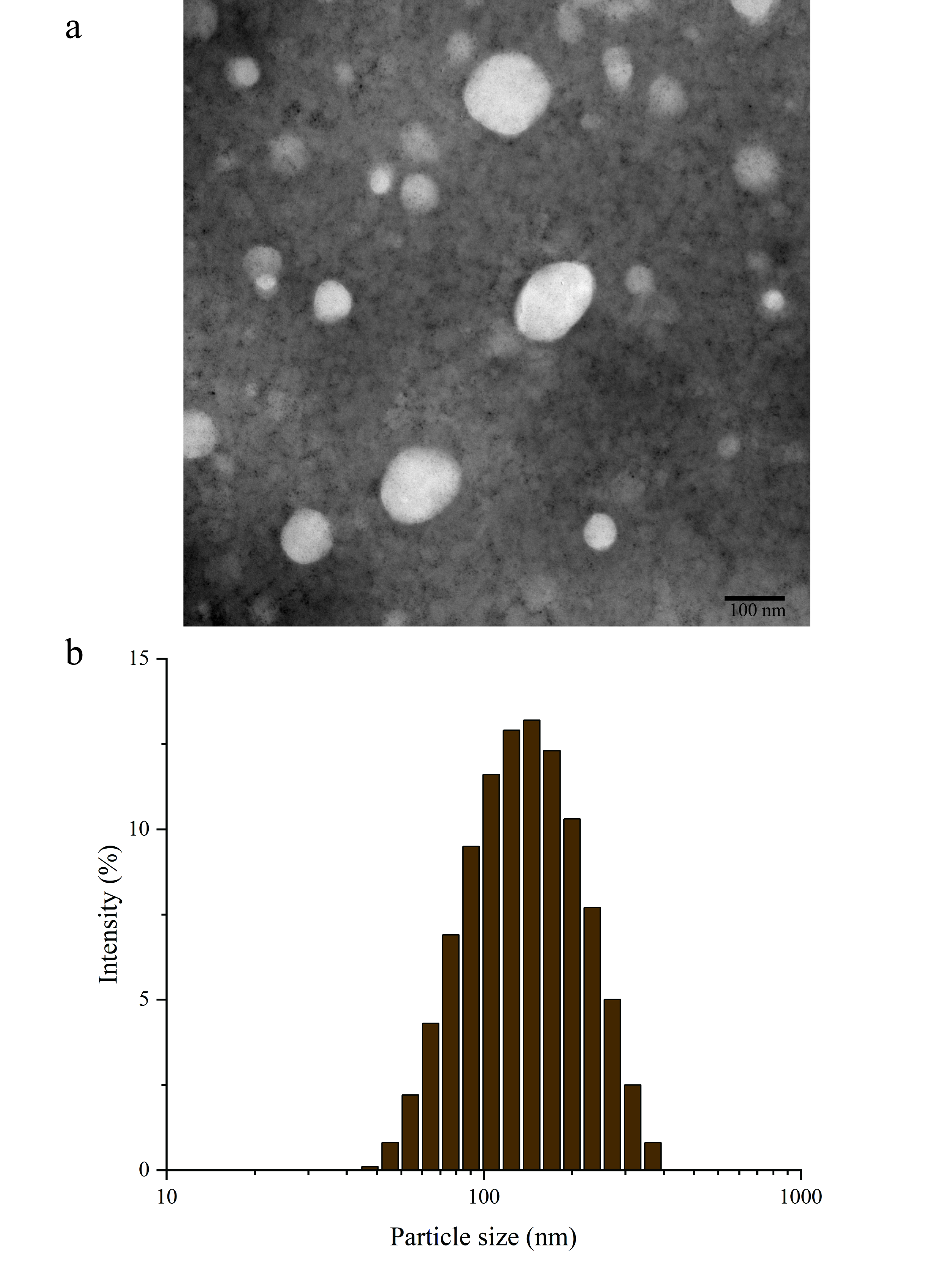

Supplement: Supplementary file 1 — Additional file 1: Figure S1. Bovine milk exosomes. (a) Electron microscopy images of exosomes isolated from bovine milk. (b) Particle size analysis of exosomes isolated from bovine milk by ultracentrifugation. [file 12864_2019_6338_MOESM1_ESM.tif]

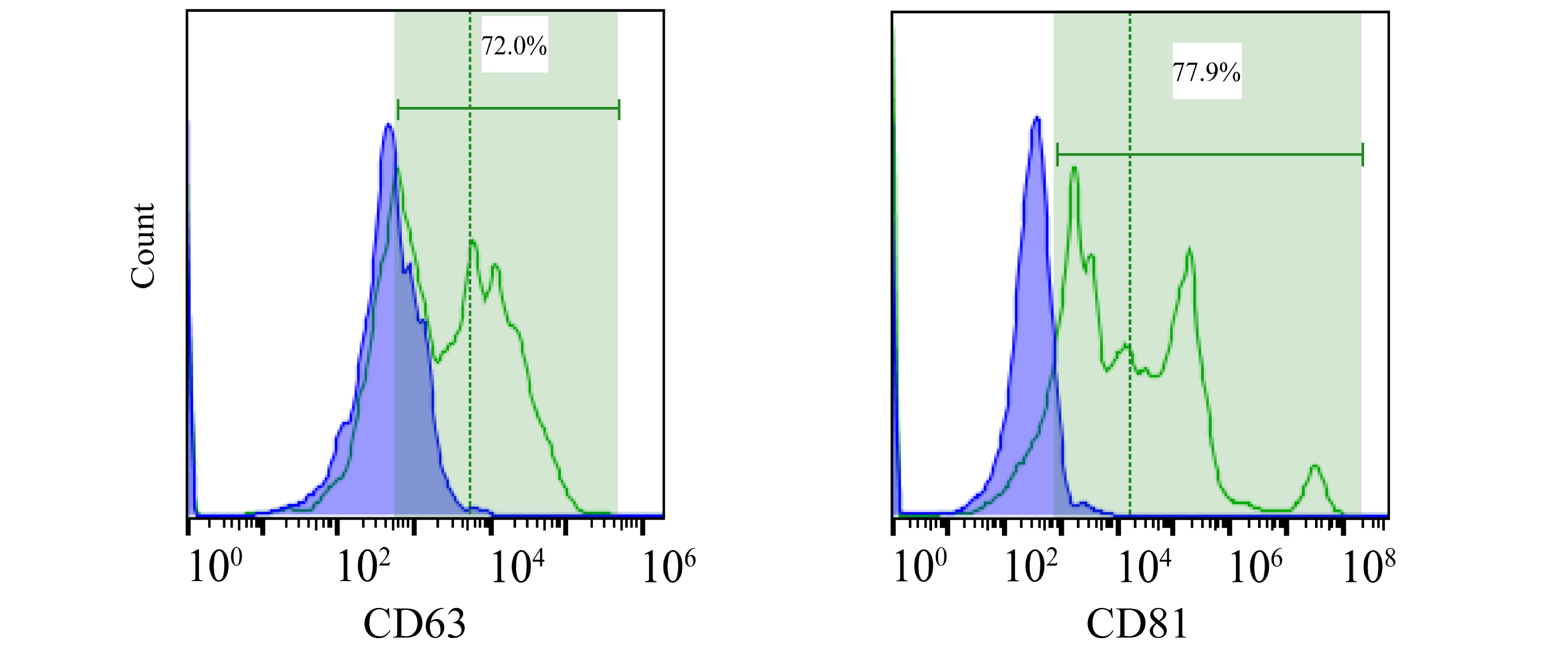

Supplement: Supplementary file 2 — Additional file 2: Figure S2. Expression of CD63 and CD81 on exosome surfaces by flow cytometry. [file 12864_2019_6338_MOESM2_ESM.tif]

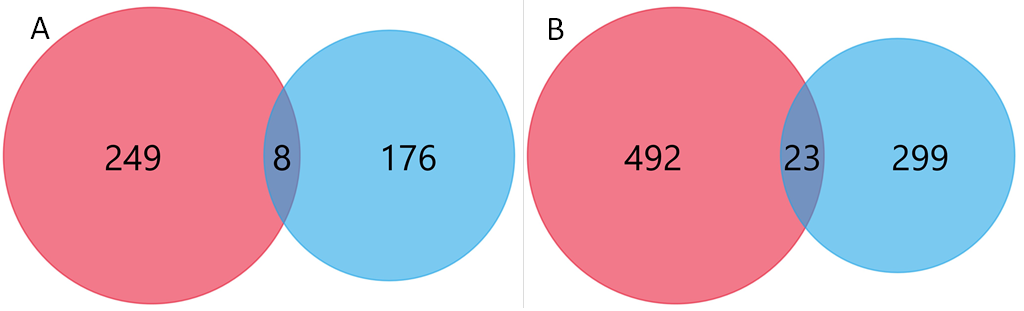

Supplement: Supplementary file 4 — Additional file 4: Figure S4. Number of predicted target genes of bta-miR-378 (A) and bta-miR-185 (B) by TargetScan (blue) and miRanda (red) programs. [file 12864_2019_6338_MOESM4_ESM.tif]

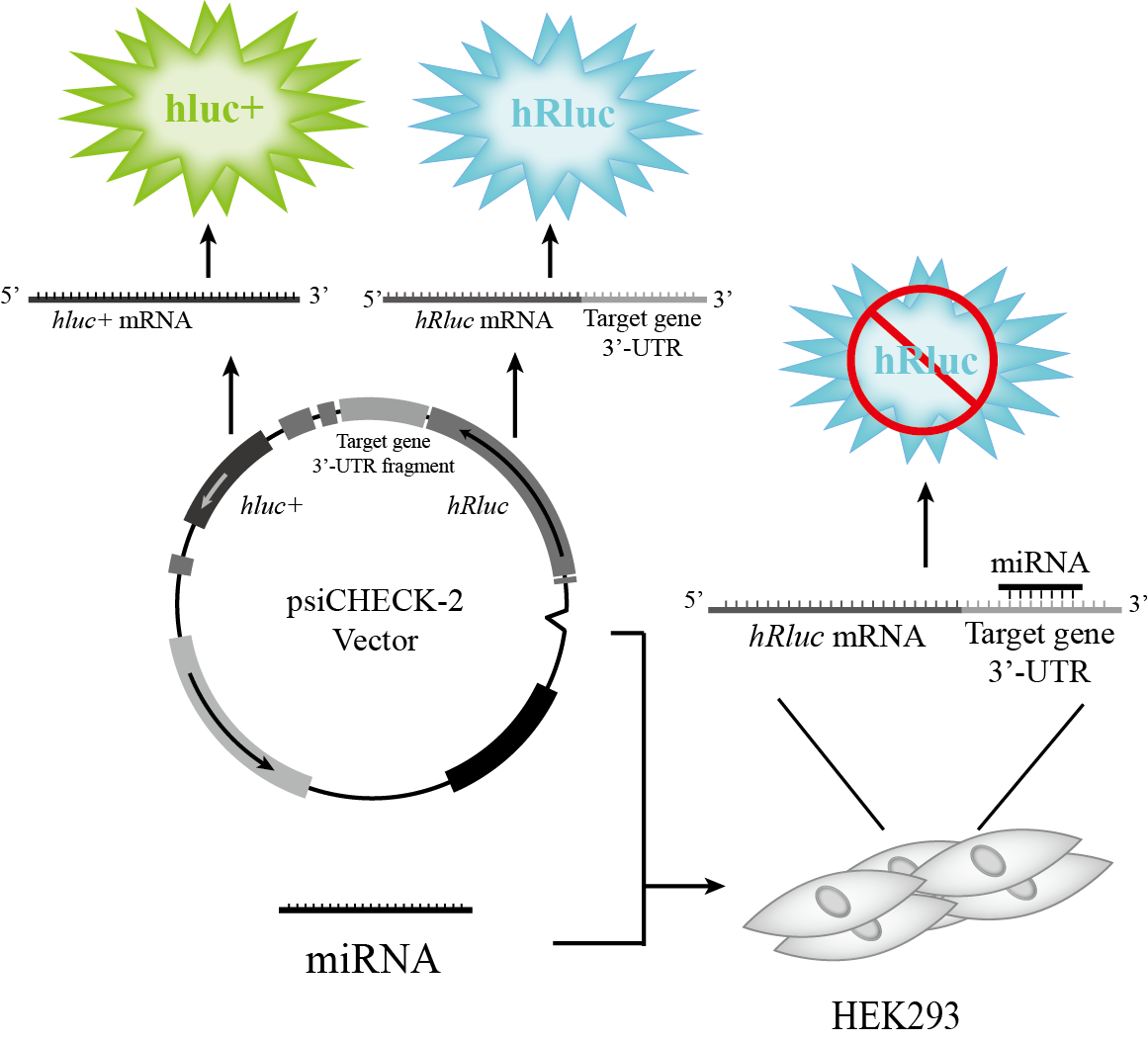

Supplement: Supplementary file 7 — Additional file 7: Figure S7. Verification of in silico predicted bta-miR-378 and bta-miR-185 targets with a dual luciferase assay. HEK293 cells were transfected with bta-miR-378/bta-miR-185 mimics together with a vector carrying the firefly luciferase gene (hluc+) as well as the Renilla luciferase gene (hRluc) fused to the 3′-UTR fragment containing either the predicted bta-miR-378/bta-miR-185 binding site or the mutated seed sequence. The assays were performed 48 h later, and the ratio of Renilla luciferase activities to firefly luciferase activities in cells transfected with an empty expression vector without 3′-UTR fragment of the target gene was set to 100%. [file 12864_2019_6338_MOESM7_ESM.tif]
